# TimeTeller for timing health: The potential of circadian medicine to improve performance, prevent disease and optimize treatment

**DOI:** 10.3389/fdgth.2023.1157654

**Published:** 2023-04-18

**Authors:** Benjamin Dose, Müge Yalçin, Sebastian P. M. Dries, Angela Relógio

**Affiliations:** ^1^ Kalms Consulting GmbH, Berlin, Germany; ^2^ Institute for Theoretical Biology (ITB), Charité—Universitätsmedizin Berlin, Corporate Member of Freie Universität Berlin, Humboldt-Universität zu Berlin and Berlin Institute of Health, Berlin, Germany; ^3^ Molecular Cancer Research Center (MKFZ), Medical Department of Hematology, Oncology, and Tumour Immunology, Charité—Universitätsmedizin Berlin, Corporate Member of Freie Universität Berlin, Humboldt-Universität zu Berlin and Berlin Institute of Health, Berlin, Germany; ^4^ Institute for Systems Medicine and Faculty of Human Medicine, MSH Medical School Hamburg, Hamburg, Germany; ^5^ Fraunhofer Institute for Software and Systems Engineering ISST, Dortmund, Germany

**Keywords:** circadian rhythms, circadian medicine, personalized treatment, health maintenance, performance optimization, diagnostic tools, computational predictive models, TimeTeller

## Abstract

Circadian medicine, the study of the effects of time on health and disease has seen an uprising in recent years as a means to enhance health and performance, and optimize treatment timing. Our endogenous time generating system -the circadian clock- regulates behavioural, physiological and cellular processes. Disruptions of the clock, *via* external factors like shift work or jet lag, or internal perturbations such as genetic alterations, are linked to an increased risk of various diseases like obesity, diabetes, cardiovascular diseases and cancer. By aligning an individual's circadian clock with optimal times for performing daily routines, physical and mental performance, and also the effectiveness of certain therapies can be improved. Despite the benefits of circadian medicine, the lack of non-invasive tools for characterizing the clock limits the potential of the field. TimeTeller is a non-invasive molecular/digital tool for the characterization of circadian rhythms and prediction of daily routines, including treatment timing, to unlock the potential of circadian medicine and implementing it in various settings. Given the multiple known and potentially yet unknown dependent health factors of individual circadian rhythms, the utility of this emerging biomarker is best exploited in data driven, personalized medicine use cases, using health information across lifestyle, care, and research settings.

## Introduction

Humans are a rhythmic species that evolved a time-keeping mechanism which allows them to adapt to environmental cycles, including day-night and feeding-fasting cycles, and to better anticipate opportunities and challenges—the circadian clock. The human circadian clock regulates a number of cellular and molecular mechanisms and plays a vital role in maintaining human health (1). Around 50% of human genes follow 24 h rhythms (circadian) in their expression in at least one tissue (2). Disruption of circadian rhythms (CR) is associated with diseases including sleep disorders, depression, diabetes, neurodegenerative diseases, obesity and cancer (3). Disruption may be caused by conflicting external or internal signals that are not in synchrony with the internal circadian time (4). Almost all biological functions are ruled by and follow a CR (5). Prominent examples are sleep–wake and feeding–fasting cycles, as well as cognitive performance, body temperature, insulin sensitivity, glucose metabolism, hormone secretion and blood pressure. The ultimate goal of such temporal organization is to keep us in balance and in synchrony with the changing environment.

The Nobel Prize for Physiology or Medicine was awarded in 2017 for the discoveries made in the past decades on the genetic and molecular mechanisms of the biological clock (5–11). Even though mounting evidence points to a crucial role of the biological clock in the proper functioning of the organism, the application of this knowledge to the maintenance of health and treatment optimization is still scarce. Published studies have shown that timing treatment based on the circadian rhythm can have a significant impact on the efficacy and toxicity of anti-cancer treatments. This approach is commonly referred to as circadian medicine or chronotherapy. The concept of chronotherapy in cancer is at least partially based on the fact that cancer cells have different circadian rhythms compared to normal cells (12, 13). Chronotherapy aims to exploit these differences in circadian rhythms by administering treatments at times when cancer cells are most vulnerable, and healthy cells are least vulnerable, thereby reducing toxicity and enhancing efficacy. Several studies reported significant reduction in side effects, such as nausea, vomiting, and fatigue and an increase in effectiveness of the treatment, leading to better outcomes and increased survival (14).

**Figure 1:**
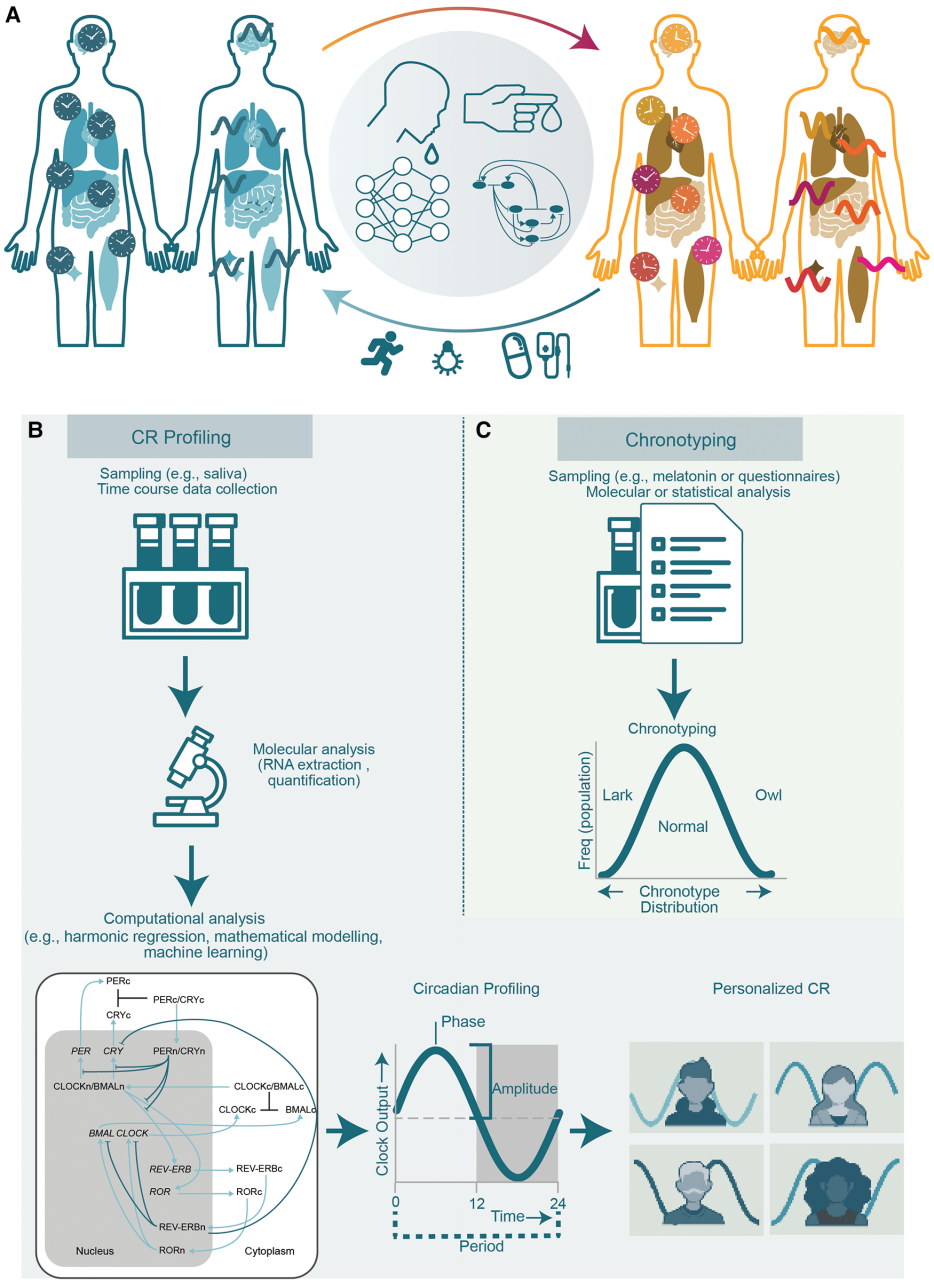
**The circadian system and methods for determining CRs and chronotyping**. (**A**) The role of the circadian clock in health and disease. A proper functioning clock ensures correct timing of physiological events such as sleep/wake cycles and contributes to sustain a healthy life (left). The disruption of CRs (right) is associated with numerous diseases including sleep disorders, neurodegenerative diseases, and cancer. Various circadian relevant clinical applications are being studied and implemented to diminish the negative effects of circadian disruption e.g., by the usage of zeitgebers like bright light therapy or physical exercise to reset the clock, and timely administration of drug compounds. For this purpose, methodologies (centre) using molecular data retrieved from time course collection of biological samples for e.g., from blood or saliva in combination with computational and mathematical modelling of the clock network are needed to generate a personalized profile of the CR. (**B**) CR profiling by repetitive and representative sampling, molecular and computational analysis to generate a personalized circadian profile. (**C**) Molecular analysis of biological samples or questionnaires and correlation to populational data for typification of an individual into chronotypes.

Despite the apparent benefits of circadian medicine, the lack of available non-invasive tools for characterizing the clock in humans limits the practical application of this field (15). The pattern of CR shows large variations and relies on several factors including: genetics, age (16), sex hormones (17), and light exposure depending on the intensity, duration, and timing of light applied (18). In the following, we provide an overview of the relevance of CRs for health maintenance and treatment optimization, as well as on the recent efforts made to develop experimental and computational tools for the characterization of CRs (Figure 1A). Finally, we will focus on a recent tool that our group has developed, which allows to profile and monitor CRs in humans and discuss its potential applications in the circadian medicine field and optimization of health. With the development of smart mobile devices and most recently wearable smart devices, biomarker and biofeedback-based lifestyle and health applications emerged, gained traction and are by now a given for significant parts of the population. This spurs a development of more granular, lower threshold health and disease management solutions and services that expand the digital health spectrum beyond remote patient monitoring. Telehealth services focused on a small number of high burden diseases to multivariate data driven approaches to personalized health and treatment management that blend a variety of person/patient generated health data.

## The endogenous biological clock

A proper functioning clock ensures the timely regulation of crucial physiological processes such as hormonal activity, core body temperature, sleep/wake cycles, immunological activity, and metabolism (19). At the molecular level, the mammalian circadian clock consists of transcriptional and translational interlinked feedback loops of 14 elements including positive regulators (CLOCK, BMAL, ROR), and transcriptional repressors (PERs, CRYs, REV-ERB) (20), resulting in a self-sustained oscillatory system. This 24 h rhythmic oscillation regulates the expression of various target genes also known as clock-controlled genes. In the main pacemaker, the SCN (suprachiasmatic nucleus), located in the brain, the expression of the gene *BMAL1* (also knowns as *ARNTL)* peaks around the middle of the active phase typically mid-day, and the expression of the gene *PER2* peaks around the middle of the resting phase, in the middle of the night, with a 12 h-shift to the peak of *BMAL1* (21). In human peripheral tissues, genes of the *PER* family peak around dawn in humans while results for *BMAL1* vary heavily across studies suggesting a higher variation than observed for *PER2* expression patterns.

The CR is generally stable over time and only adapts to seasonal changes due to the fluctuation in light exposure (22) or can be disturbed by profound and lasting fluctuations of daily routines. While the CR is robust to alterations like temperature fluctuations, the re-alignment of the CR to a new-time zone is not immediate and may require several days to adjust commonly referred to as jet lag (23). Today, with the help of extensive research at the molecular level, it is known that the CR does not simply regulate sleep-wake cycles, but also influences numerous cellular processes, including DNA damage response, metabolism, and cell proliferation (24). This knowledge allows for a range of potential clinical applications including optimization of daily activities such as physical exercise, or light exposure, and therapeutic administration of drugs considering the CR of the individual and the drug (Figure 1A).

## Circadian regulation, dysregulation and adjustment: time matters in health and disease

The circadian clock influences and times important functions in our bodies, including hormone release, eating schedules and digestion, body temperature and sleep. In humans, the clock can be adjusted by various external signals. These timing cues, also called zeitgebers (external or environmental cue that synchronizes the circadian clock), can reset the circadian clock. The timely usage of zeitgebers during the day allows for the maintenance or reestablishment of a robust CR and improves health and quality of life (25). The multitude of dependent health effects and the high impact of the CR on everyday life warrants a digital twin approach for people to understand and give them control over utilization of the CR and (potentially) dependent data. As there is not just one CR, but different individuals and health vs. disease status express different rhythm patterns, the generation of computational models of its interdependence with lifestyle, health and care effects will evolve over time. Extending beyond sensor-based measurements of common bio-signals like pulse, blood oxygenation, motion and derived measures, CRs will likely be included in future parameter sets of personal health and fitness dashboards as for e.g., included in common smart device platforms. Users, no matter if they are just interested people or patients under treatment, can be provided with CR data based on initial and potentially repeated individual calibration from for e.g., saliva sampling or wearable sensing. Service providers that can be healthcare providers, payers, or health IT solution/device/drug vendors or combinations of these can offer research, lifestyle, or clinical decision support services for e.g., based on computational models that correlate personal health events and signals, treatment (scheduling) data and CR for upstream evidence generation and downstream clinical decision support. In the following, we provide a brief overview of physiological and behavioural aspects that are CR-dependent (sleep, aging) and/or influence the CR (light, meal times, exercise) and can thus be used as zeitgebers, to enhance or even shift the CR, in case of a dysregulation.

### Circadian rhythms and aging

An interesting, and particularly relevant application of enhancing the CR relates to aging. Aging is characterized by a continuous decline in physiological and molecular processes (e.g., endocrine, behavioural and locomotor activities) and results in increased susceptibility to aging-related diseases such as neurodegenerative diseases and cancer (26, 27). Aging may lead to circadian alterations accompanied by sleep disturbances, and reduced cognitive performance (28). Pre-clinical studies on aging animal models have shown a decline in the phase and amplitude of core clock genes such as *PER2* and *BMAL1*. Thus, a healthy CR may exert profound influences on whole-body rejuvenation and the appropriate usage of zeitgebers may support it. Stimuli including social interaction, physical exercise, stress and caffeine can all act as clock synchronizers in the body (29).

### Circadian regulation of sleep

The circadian system regulates the timing of sleep and wake up and aligns the human physiology with it. During sleep several important functions take place including clearance of toxins, cellular repair, memory consolation and information processing (30). Furthermore, sleep has been shown to improve the mood, alertness and concentration, boost the immunity and reduce the risk of other chronic diseases such as heart problems (31). Continuous exposure of the CRs to conflicting zeitgebers like artificial light for example due to night-shift work or jet lag can result in disruption of sleep thereby affecting these processes (32).

### Light therapy against circadian dysregulation

Light is one of the strongest zeitgebers, which can align the period of two or more oscillators (for example, the SCN to the environmental light/dark cycles) a process known as entrainment (33). Unlike entrainment, synchronization does not require oscillators, but rather aligns the phases throughout the body. Environmental light is perceived *via* intrinsically photosensitive retinal ganglion cells (ipRGCs) in the retina. The information is then transmitted to the central pacemaker in the brain (SCN), entraining it to a 24 h solar day. Subsequently this information is transmitted to the rest of the body *via* humoral and autonomic nervous system signals. Mistimed light exposure may lead to misalignment of CRs and negatively impact human health, being associated to mood and sleep disorders, neurodegenerative diseases or cancer (34). In recent years, light therapy has been increasingly implemented as an assisting/supportive therapy for various medical conditions, from depression and sleep disorders (35) to cancer (36). For cancer patients, light therapy has proven to help overcome side effects of the treatment such as treatment-related fatigue. Recently, new applications of light therapy are emerging in internal medicine, especially relevant in intensive care units where artificial light differences between day and night can be minimal. This may result in patients developing a fragmented sleep-wake cycle with a negative impact on their recovery and wellbeing, or even cause delirium (37).

### Feed/fasting cycles as synchronizers of the circadian clock

Also feed/fasting regimes can be used to synchronize the circadian clock (35). Biological processes like metabolism are closely linked to our CR (38). Meal time is an important factor leading both to circadian disruption or regulation. Peripheral CRs, like in the liver, are more profoundly affected by the timing of food intake than the timing of light (39). Previous studies on humans and animal models suggested that the consumption of a larger portion of caloric intake during the first half of wakeful hours may be preferred for better blood glucose regulation and weight control (reviewed in (40)). In addition, caloric restriction that involves time-restricted feeding of less than 6 h has been suggested to improve longevity in mammals (41, 42). Therefore, consolidating eating periods to defined intervals (so-called time-restricted eating) is another behavioural intervention that can support robust CRs in peripheral organs and improve health.

### Circadian dependence of physical activity

Finally, also physical exercise is both influenced by the clock and can act as a clock enhancer and synchronizer. Several physiological features which influence sports performance show circadian variation, including core body temperature, hormone levels, blood pressure, and heart rate variability (43). Core-clock genes (e.g., *BMAL1*), influence skeletal muscle function. Physical exercise is an important enhancer of the CRs. Several studies point to a daily variation of exercise performance. Our group also previously reported a correlation between *PER2* peak timing and strength performance whereas amplitude of *BMAL1* seems to correlate with overall daily variation of exercise performance (44). Thus, the personal CR can be used to determine the variation of athletic performance throughout the day and predict the optimal time window for peak athletic performance. The extent and duration of the effect depends on the intensity and duration of the exercise (45).

### Regulation of circadian rhythms

A well-functioning circadian clock, in synchrony with an individual's behavioural habits, can improve general health and wellbeing, will improve overall fitness, and reduce recovery or therapy time in patients. To avoid or overcome CR disruption, a person's individual internal rhythm can be profiled and used to adjust relevant external/internal factors. The efficiency of activities such as sleep, sports, or medicine intake can be optimized based on the internal timing. Interventions such as light therapy, exercise, and shifting the individual rhythm pharmacologically (for example with melatonin supplements) have been used in recent research to revert the effects of disrupted CRs (46). A disruption of normal CRs (e.g., due to constant shift work or genetic disruption of clock mechanism) has been shown to negatively affect an individual's wellbeing, metabolism, physical and cognitive performance and may lead to disease including sleep disorders, obesity, depression, neurodegeneration and cancer as it facilitates tumorigenesis and the establishment of cancer hallmarks (47, 48). Cancer cells can be arrhythmic, rhythmic in-phase or rhythmic out-of-phase with healthy tissues (14, 49) (e.g., metastatic spread of breast cancer accelerates during sleep) (50). Altered circadian rhythmicity in cancer cells allows for chronotherapy with the following rationale: identification of a timepoint of lowest toxicity to non-cancer cells, at which the maximum tolerated dose can be administered to impact tumour cells, while sparing healthy cells, for a given patient patient. The severity of side effects could be reduced by CR-based treatment schedule optimization. A proof of principle was recently published by our group showcasing a mathematical model of the circadian clock and drug pharmacology to optimize irinotecan administration timing in colorectal cancer to reduce its toxicity (51). Proper timing of chemotherapie to increase drug-tolerability is particularly relevant for anticancer drugs, as they are often administered close to their maximum-tolerated dose (52, 53).

## Measuring CR: molecular and physiological measurements

Various approaches can be used to investigate circadian rhythmicity in humans both in research, as well as in a clinical context. However, a clear distinction of their 1) information value (a) CR vs. outputs of the CR; b) chronotyping vs. CR profiling) and 2) usability are extremely relevant to consider (see Table 1).

**Table 1:** Selection of commercial and scientific tools for circadian clock assessment in humans. *Sleep recommendations.

	BODYCLOCK®Hairtest	Geneplanet®*	Cerascreen®Melatonin Test	Salimetrics®Salivary DLMO profile **(****54****)**	WEARABLES/mobile phone apps**(**55****)**	Munich ChronoType Questionnaire (MCTQ) **(****15****)**
**Sample material/method**	Hair follicle (at least 15)	Saliva	Saliva	7 saliva samples	Light reflection, spatial acceleration, electric conductance,	Questionnaire
**Sampling protocol**	Invasive, single time point	Single time point	Single time point	Non-invasive, multiple time points (within 7 h)	Triggered/interleaved/continues	Review of last four weeks
**Target analyte**	mRNA expression level	Genomic DNA Gene Variants	Hormone concentration (Melatonin)	Hormone concentration (Melatonin)	Temperature, blood pressure, heart rate, blood oxygenation, respiratory rate, acceleration, and derived measures	Not applicable
**Result**	Qualitative determination of a chronotype based on gene expression (Note: mRNA values not provided)	Qualitative lifestyle predictions based on gene variants	Quantitative measurements of melatonin concentration from saliva over time	Quantitative measurements of melatonin concentration from saliva over time	Quantitative and/or qualitative measurements of sleep-wake pattern, activity patters	Qualitative determination of Chronotype based on the query
**Risk/Pain of procedure**	None/Somewhat painful	None/None	None/None	None/None	None/None	None/None
**Legal status**	Lifestyle product	GDPR compliant and ISO 27,001 certified	DIN EN ISO 13,485, EC declarations of conformity & interlaboratory test	Research use only	Lifestyle product, medical devices	Research use only
**Customer benefit**	Generic recommendations with respect to the chronotype	Recommendations regarding sports, nutrition, stress, and sleep/determination of chronotype	Generic recommendations for action and approaches for proper nutrition	Determining DLMO is relevant in diagnosing CR-related sleep issues	Visualization of diverse body parameters for various purposes	Estimation of chronotype mainly for research use

** e.g., rings (Oura), bands (Halo, WHOOP, Fitbit), watches (Withings, Apple, Garmin).

1a) Measurements of cortisol, analysis of genomic DNA or transcriptome, often are invasive and measure single time points to assess chronotypes (e.g., early bird vs. night owl) (54). Generic chronotherapy (e.g., morning vs. evening drug application) considers neither the CR of the individual nor of the drug activity. 1b) Core body temperature, rating scales such as morningness-eveningness questionnaire or actigraphy (15) (e.g., *via* rings (Oura), bands (Halo, WHOOP) or watches (Withings)) measure outputs of the CR not the CR itself (56). Thus, the approaches described under 1a) or 1b) are not suitable for individualised chronotherapy. 2) Methods like the Dim Light Melatonin secretion Onset time (DLMO) profiling often require an overnight stay at the clinic, are time-consuming and interfere with the sleep time of the test person, and are thus unrealistic for routine clinical use (57).

There is currently no user-friendly (non-invasive, cost-effective) and practicable method on the market (lifestyle or research use only (RUO) products) that would allow to model the CR based on the expression of core-clock genes (57) and thus be useful to advance the field of circadian medicine.

Wearable device-based measurements and derived parameters and models have evolved from specific purpose devices and platforms to multi-purpose devices, platforms and ecosystems. Whether wearable devices have the potential to accurately and consistently capture changes in the CR over time, remains to be demonstrated as wearables can only measure the outputs of the CR like body temperature, heart rate and activity, but not the underling factors like the circadian gene and protein expression network that generates these circa 24 h rhythms. One of the benefits of wearable devices is that they provide continuous and long-term monitoring of CR outputs, which may reveal alterations that might not be apparent during shorter periodic measurements of genes and protein expression.

To date, the most prevalent smart device OS platforms, Google Android and Apple iOS offer health data capture, exploration, management, integration and utilization capabilities through Google Fit and Apple Health that interface with a breadth of vendors and signal modalities. In addition, interoperability platforms and services exist that facilitate combination of signal and device modalities beyond single platform integration. The future will likely bring open, trusted, federated, interoperable and data sovereign personal health data ecosystems, as mandated in the EU by the European Health Data Space regulation. Already today, individuals can choose from a variety of apps and wearable devices to monitor combinations of bio-signals and obtain analytic insights into sleep quality in correlation to habits, as well as derived personal lifestyle and health advice as to optimize sleep quality, for instance.

## The need for a non-invasive in-vitro diagnostic for CR-profiling: TimeTeller

To allow for CR consideration a specific tool is needed that ideally is 1) non-invasive to allow easy access without ethical concerns, 2) user-friendly so that no specialized medical personnel is required, 3) can be performed at home to keep the time commitment for participants low and increase compliance, 4) provides informative output with recommendations, which are simple and easy to follow, 5) allows for monitoring *via* repetitive analysis, 6) ideally certified and approved for medical purposes to be accessible not only for research, but also in the healthcare sector.

**Figure 2:**
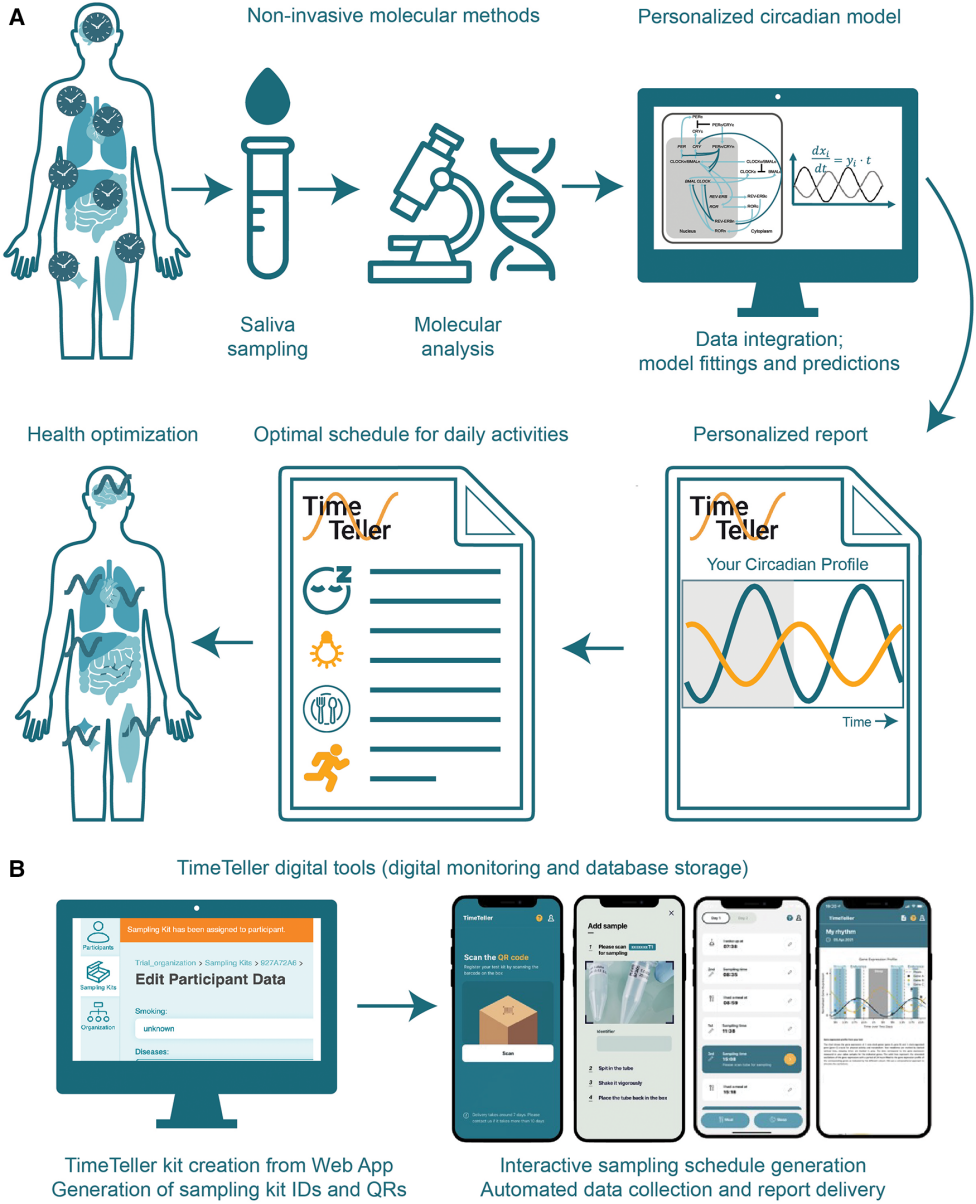
**TimeTeller: a non-invasive method for profiling the CR and prediction of optimal times for daily activities.** (**A**) TimeTeller pipeline for saliva collection at defined times, molecular and computational analysis and report generation with circadian profile and recommendations to optimize health. (**B**) Digital tools to manage sampling logistics (left) and digital patient support to increase compliance (right).

In the following we describe “TimeTeller” as an *in vitro* diagnostic (IVD) tool with the possibility to integrate CR profiling in any scientific field and question (Figure 2). The TimeTeller IVD allows to profile the individual CR in a non-invasive and user-friendly way, which can be carried out at home and does not require neither medical supervision nor assistance. It is based on saliva samples, which are collected over a period of 48 h, only during daytime with a four-hour sampling interval, in a total of 8 samples (44, 51). The collected saliva samples can be kept at room temperature for a few days (or 4 °C for several weeks) for subsequent molecular analyses (Figure 2A).

Molecular characterization of the expression of core-clock genes (e.g., *BMAL1*, *PER2*) are used as inputs for profiling and modelling the circadian clock using computational algorithms suitable to analyse circadian time series data such as harmonic regression and mathematical models (Figure 1B) (44). The profile of a person's CR defined by amplitude, period, phase, mean level (mesor) can thus be generated by measuring gene activity *via* RNA quantification in saliva samples and using computational circadian models (44). However, the accuracy of saliva-based methods can be affected by factors such as the timing of sampling, the consumption of food and drinks, and the use of medication.

For more detailed predictions for example, related to the determination of cytotoxicity times for a certain drug and patient, mathematical models based on ordinary differential equations can be used to mathematically represent the molecular processes such as transcription, translation, post-translational regulations and degradation processes of the core-clock network (Figure 2A) integrated in the metabolic network of a given drug (51). The addition of recommendations for sleeping, exercise and meal times provides a holistic picture of the personalised CR (1, 51, 58). Additional web and mobile applications allow for a followup of the sampling collection and processes steps and support patient compliance by generation sampling reminders (Figure 2B).

Significant changes to daily routines for example the disruption of sleep patterns due to jetlag or light exposure, will be reflected in the CR profile. Furthermore, perturbations or misalignment of the CR and daily routines that may manifest among others in sleep disorders can be identified (23).

In comparison to the previously available methods for circadian clock assessment (Figure 1C and Table 1), the TimeTeller tool is more comprehensive since it provides a quantified circadian profile characterised by an amplitude, peak and phase based on gene expression data of selected clock genes. Furthermore, it is non-invasive and does not require medical assistance. In addition, the informative profile of individual CRs have several, highly relevant, future health-related clinical and non-clinical applications like 1) monitoring disease progression (bio-marker), 2) optimizing medical treatment timing according to drug targets and 3) optimizing performance/efficiency by improving sports performance, or sleep quality. Hence TimeTeller may be applied to optimize treatment timing in patients, as well as for optimizing physical and cognitive performance in healthy individuals.

## Applications from health prevention to treatment timing

Based on CR-profiles determined by TimeTeller, computational tools can generate personalized predictions relevant to health, wellbeing and physical performance, as well as drug metabolism and ultimately suggest optimal times for daily activities like sleep (59), sports (44), light exposure, and medicine intake (1) (Figure 1).

TimeTeller can be used to profile the CR and identify perturbations or misalignments of the CR not only in healthy (44), but also in individuals suffering from different types of pathologies e.g., Parkinson's disease (60–62), type 2 diabetes (63), cancer (36, 64–66), depression (67). Based on the profiled CR, behavioural recommendations related to the timing of feeding-fasting (68), physical exercise (69), sleep-wake (70–72), or light-dark cycles (18), can be provided to re-align the CR with the environment of the individual. Changes to CR in response to sleep-(71, 72) and meal-timing (68) can be monitored *via* core-clock gene expression using the TimeTeller technology.

This increases well-being and diminishes certain symptoms that are typically associated with the above-mentioned diseases and/or their treatment: fatigue, sleep disorders and CRs disorders. These symptoms of circadian dysregulation are recognized as a disease by the International Statistical Classification of Diseases and Related Health Problems 10th Revision under (ICD-10-CM Code: G47: Sleep disorder/G47.20: CR sleep disorder). This is particularly relevant as the CR differs for each of us and generic recommendations do not take individual variations into account (17, 59).

### Clinical application of CRs: drug development

The overall failure rate in drug development is 96%, and 90% or higher, during clinical development (73). High attrition rates can be seen in every phase of drug development or therapeutic area and have remained at a similar level over the last few decades (74). 40%–50% of compounds fail due to lack of clinical efficacy while 30% show unmanageable toxicity (73). Expanding the drug developmental scope by considering the dimension “time” has the potential to significantly improve these statistics (48).

Chronotherapy uses the circadian rhythmicity (CR-dependencies) of cellular processes in the administration of treatment to maximize effectiveness and minimize side effects in drug development and treatment. To this end, treatments for a broad range of medical conditions and their links to the CRs have been investigated including, but not limited to: allergy, arthritis, asthma, hyperlipidaemia, hypertension, cancer, and neurodegeneration (48). The therapeutic relevance of the circadian clock gained an increased attention in recent years. One reason being that 56 of the top 100 best-selling, FDA approved drugs in the United States, target genes that are expressed in a circadian manner, including all top 7 best-selling drugs (USA) (75). This is expected as a total of 43% of all protein-coding genes show CRs (75). Additionally, nearly 50% of the top 100 drugs also have a half-life time of less than 6 h, an important requirement to manage the drug concentration in the body in a timely defined manner (75). Of note, a study that screened over 1,000 molecules from an FDA-approved drug library found that approximately 5% of the drugs screened altered the circadian period (76). However, currently, the CR does not play an adequate role in drug development, even though the attrition rate, defined as the loss of new candidate drugs during the process from pre-clinical to clinical and through their clinical development, is on a high level. Multiple phase III clinical trials testing chronotherapy vs. conventional treatment schedules showed an improved anticancer treatment tolerability of up to fivefold and a nearly double efficacy in experimental studies (77, 78). Similar chronopharmacological effects have been shown for various drugs (77). A recent systematic review reported that 61% of analysed studies described significant decrease in toxicity of chemotherapy (14). Considering this body of knowledge, the CR has the potential to become an essential clinical parameter in drug development to reduce high attrition rates.

Along with the development of stratified, precision, or even individualized medicine, data driven approaches to drug development, real world evidence generation, and clinical decision support emerge and will shorten cycles, as well as improve targeting of selection, dosing, and potentially even timing based on chronobiology. Data ecosystems that span people, care providers, and in some constellations pharma and/or payers will scale up such that real time treatment management becomes available to everyone in need, while safeguarding data utilization within the stated purpose, by the authorized roles and for the agreed period. Dynamic and adaptive treatment schedules that make time-dependent interactions between drugs, individual physiology, and diseases manageable for individuals and their care providers will gradually replace the “x times daily” type of current intake schedules.

## Discussion

With the recent advances in methods for molecular analysis techniques and the development of digital tools, circadian considerations are expected to become an integral part of healthcare and disease management. By focusing on the internal biological clock, which governs the timing of everyday behaviours, circadian medicine has the potential to revolutionize the way we approach healthcare.

A combination of molecular analysis and digital tools can be used to characterize and monitor the CR, but there is still a need to develop user friendly tools that can make this process easier. In the future, tools like TimeTeller, which allow for a full characterization of the CR, will allow to adjust daily routines to optimize health and improve treatment efficacy. The CR specifically warrants research into how an initially identified CR pattern will be carried forward using continuous or spot check measurements, and into hypothesis generation and validation through interoperable, transparent and data sovereign utilization of CR and associated data across lifestyle, care, and research settings using machine learning approaches.

## Conclusion

As personalized medicine continues to advance, it is essential for clinicians and researchers to recognize the significance of the CR and to have tools available to measure and monitor it, both to detect possible CR dysregulations that can act as a potential biomarker for diagnostics, and to design individualized treatment plans tailored to the specific needs of each patient. For example, lifestyle modifications such as altering timings of meals, and physical exercise may be recomended to a patient with sleep disorders to achieve a healthier sleep cycle. Similarly, adjusting the patients' treatment administration schedule based on their CR may enable a reduction of side effects and an increase in treatment efficacy. The future of personalized medicine looks bright, and circadian medicine has the potential to make a real difference in patients' lives. Time matters and it's time to use it!

## Data Availability

The original contributions presented in the study are included in the article/Supplementary Material, further inquiries can be directed to the corresponding authors.
